# Biogas production and microbial profile estimation in bioreactor landfills

**DOI:** 10.3389/fchem.2026.1742729

**Published:** 2026-04-07

**Authors:** Yunmin Zeng, Shulin Pan, Aiping Zhang, Jianrong Wang, Fan Huang, Jian Yu, Qiang Luo, Bing Ren, Walid Tahri

**Affiliations:** 1 Faculty of Quality Management and Inspection and Quarantine, Yibin University, Yibin, China; 2 Key Laboratory of Special Waste Water Treatment at Sichuan Normal University, Chengdu, China; 3 Yibin Vocational and Technical College, Yibin, China; 4 Sichuan Tongyi Environmental Science and Technology Group Co., Ltd., Yibin, China

**Keywords:** anaerobic digestion, bioreactor landfill, leachates, methane yield, microbiome

## Abstract

**Introduction:**

This study investigated the municipal solid waste (MSW) biodegradation process, simulating landfill conditions using a bioreactor. A core objective was to identify key markers in leachate that could forecast the methane (CH_4_) generation process during anaerobic digestion (AD). To further understand the causes of CH_4_ production inhibition and to propose strategies for enhancing AD system performance, we aimed to compare the microbial community structures in leachate from different reaction periods and in solid MSW samples.

**Methods:**

A bioreactor was utilized to replicate the landfill’s MSW biodegradation process. Research workers analyzed the relationship between the methanogenic process and the properties of leachate from anaerobic digestion. To investigate the underlying causes of inhibition, we compared the features and differences in the microbial community structure of leachate samples from different reaction periods and solid samples (end-state MSW and cover layer).

**Results:**

The biogas production potential was found to be 74.36 L kg^−1^, and the rate constant for MSW digestion gas production was 0.0359 days^−1^. A correlation was observed between the leachate’s pH, TOC/TN ratio, and the CH_4_ generation process, though the correlation between pH variation and methanogenesis showed a clear lag, indicating pH alone is not a sufficient predictive signal. The system became unstable due to ammonia buildup, with a TOC/TN value below 13 coinciding with minimal gas output. Microbial analysis showed that the genetic similarity between leachate and MSW samples was inversely related to the length of the reaction period. A key observation was the absence of Nitrospirain leachate, which likely interrupts the nitrogen conversion cycle. The nitrification process was found to primarily occur in the cover layer. Decreased CH_4_ generation was mostly caused by ammonia inhibition, which reduced the activity of acetate-utilizing methanogenic archaea. The intermediate cover layer acted as a biochemical reaction zone with greater microbial diversity.

**Discussion:**

The findings indicate that due to ammonia buildup, the fermentation system became unstable when the TOC/TN value fell below 13. The absence of Nitrospirain leachate is identified as a critical factor disrupting the nitrogen cycle. Therefore, inoculation with Nitrospira-containing agents is proposed as crucial for maintaining system stability and enhancing treatment efficiency. The intermediate cover layer, harboring greater microbial diversity, contributed to enhanced anaerobic digestion and supported increased system stability, functioning as a vital biochemical reaction zone. These insights provide recommendations for enhancing the AD system’s CH_4_ production capacity.

## 1 Introduction

The treatment of municipal solid waste (MSW) in China is primarily accomplished through properly maintained landfills (Zhang et al., 2010a). One of the most important recoverable energies is methane (CH_4_), which comprises approximately 40%–60% (Broun and Sattler, 2016) of landfill gas (Aydi et al., 2015; Broun and Sattler, 2016; Di Maria et al., 2014). The bioreactor landfill method has been suggested as a solution to the problems caused by conventional landfills, such as their slow deterioration and limited biogas generation. The organic components in MSW can be degraded and stabilized more swiftly by introducing biofortification procedures into anaerobic digestion (AD). These options include nutrient addition, pH and temperature management, oxygen delivery, microbial inoculation, and leachate recirculation (Aguilar-Virgen et al., 2014; Nikolaou et al., 2010; Reinhart et al., 2002; Townsend et al., 1996; Wang and Pelkonen, 2009; Yang et al., 2006).

Online monitoring and real-time regulation are crucial for maintaining the steady-state operation of AD systems and enhancing energy recovery efficiency. However, in practical engineering applications, there are multiple challenges to obtaining comprehensive and representative indicators that accurately reflect the waste degradation status. First, the inherent spatiotemporal heterogeneity of waste composition leads to complex and highly variable degradation behaviors. Second, establishing a monitoring network involves substantial capital investment and ongoing maintenance costs, which limits its large-scale deployment. Third, the harsh conditions within landfill bodies make it difficult to collect spatially representative samples. Finally, data obtained from localized sampling often lack representativeness, hindering accurate extrapolation to the overall degradation process. These factors collectively constrain precise process control and timely system regulation.

Leachate from landfills, a liquid by-product generated during the AD of waste, is closely linked to the decomposition dynamics and CH_4_ yield of the waste. It not only indicates the extent of organic matter degradation but also directly influences the stability of landfill systems and their associated environmental impacts. Consequently, key leachate properties, including pH, chemical oxygen demand (COD), volatile fatty acids (VFAs), ammonia–nitrogen concentration, and production volume, are regarded as critical indicators for assessing the progress of landfill stabilization. By systematically monitoring metabolite concentrations and microbial community structure in leachate, it is possible to dynamically predict and evaluate the stages of AD without disturbing the internal waste environment. This approach offers a cost-effective, efficient, and noninvasive strategy for monitoring waste treatment processes. The concentrations of COD and VFA change according to the level of stability of the waste (Nair, 2013). On the other hand, Luo et al. (2004) illustrated that the amounts of biochemical oxygen demand (BOD) and COD in wastewater can be used to determine the fermentation stage of MSW. The concentration of hydrogen, the pH of the liquid phase, and the concentration of CH_4_ were the critical control parameters in the anaerobic fluidized bed bioreactor that Moletta et al. (1994) created. The possibility of using volatile fatty acids in biogas slurry as technical indicators in the AD of substrates based on dung and urine was assessed by Ahring et al. (1995). In a study on stability control during the anaerobic fermentation of vegetable waste, Meng et al. (2011) identified a key early-warning indicator for system acidification and instability: the concentration of VFAs, particularly the propionate-to-acetate ratio. This parameter is more sensitive and provides earlier warning than traditional measures such as pH because VFA accumulation directly reflects the microbial metabolic imbalance and occurs before a significant decrease in the pH. In practice, monitoring this ratio allows operators to implement corrective measures such as reducing the feeding load or adding a buffering agent several days in advance, thereby maintaining process stability and improving biogas production efficiency.

Key leachate parameters, such as pH, total organic carbon (TOC), total nitrogen (TN), and VFAs), were continuously measured during mesophilic AD digestion of MSW in a bioreactor system and gas generation. Furthermore, in order to find useful markers for tracking CH_4_ production progress and keeping the system stable, we observed the trends over time of these indicators and how they were interrelated. The results offer technical backing for optimizing and adjusting the operating parameters in real time. In addition, studying the microbiome has shed light on the biological processes driving CH_4_ production, which in turn has provided information that can assist bioreactor landfills in producing more biogas.

Moreover, in order to evaluate gas generation and leachate composition, important parameters (e.g., pH, TOC, TN, and VFA) linked to CH_4_ production were monitored during the mesophilic AD of MSW in the bioreactor. The real-time optimization of the operational conditions was facilitated by analyzing the trends and interrelations among various factors, enabling the identification of dependable metrics for predicting the CH_4_ yield and ensuring overall system stability.

## 2 Experimental procedures

### 2.1 Materials

A MSW transport station served as a garbage collection facility in the Shapingba area of Chongqing, China. Based on the Sampling and Analysis Methods of Domestic Waste (CJJ134-2009), the findings of the MSW content analysis are summarized in Table 1. Furthermore, an inoculated sludge, defined as anaerobically active sludge and used to provide microbial consortia for reactor start-up, was collected from the wellhead sewage treatment plant operated by Shapingba Drainage Co., Ltd. (Chongqing, China). As a biological medium, inoculated sludge contains a large number and wide variety of microorganisms. Mixing the inoculated sludge with waste can accelerate early-stage MSW degradation, reduce the accumulation of hydrolysis and acidification products that inhibit methanogens, and shorten the proliferation period of dominant microorganisms. The leftover dirt from demolished buildings on the Chongqing University campus was used as the foundation for the intermediate cover. Table 2 presents the detailed chemical and physical characteristics of the cover soil, inoculation sludge, and solid waste. Statistical differences between the characterized materials were determined by one-way ANOVA, with a significance threshold of p < 0.05.

**TABLE 1:** MSW content.

MSW	Food waste	Fruits and vegetables	Paper	Textile	Wood	Plastic	Spodosol rocks	Brick, tile, and ceramic	Glass	Metal
Rate	50.15 ± 2.43	22.82 ± 1.77	9.34 ± 1.02	3.16 ± 0.85	1.91 ± 0.16	8.40 ± 2.48	1.48 ± 0.14	0.92 ± 0.02	1.46 ± 0.27	0.36 ± 0.03

**TABLE 2:** Simulated characteristics of MSW samples, sludge, and soil.

Characteristic	MSW	Sludge	Soil
Density (kg m^-3^)	351.19 ± 3.48	1 000.00 ± 12	1 083.00 ± 7
Moisture (% wet wt.)	54.39 ± 2.84	79.07 ± 2.91	5.34 ± 0.68
Volatile solids (% dry wt.)	85.47 ± 4.27	40.49 ± 1.83	13.49 ± 1.13
C (%)	43.15 ± 0.34	20.49 ± 0.17	8.49 ± 0.11
H (%)	4.86 ± 0.05	2.45 ± 0.02	0.33 ± 0.02
N (%)	2.43 ± 0.07	3.32 ± 0.09	0.58 ± 0.01
S (%)	1.07 ± 0.02	1.11 ± 0.02	0.40 ± 0.01
C/N (%)	17.86 ± 0.10	6.18 ± 0.07	14.58 ± 0.09

### 2.2 Preparation of cover soil

The soil that was utilized for this experiment was allowed to air-dry before being screened through a 20-mesh sieve. To create the modified intermediate covering soil, 5% lime and 0.1% polyacrylamide (PAM) were mixed into the soil. Prior research has shown that modified covering soil has desirable engineering properties, such as the ability to neutralize organic acids generated by anaerobic fermentation of waste. This helps to prevent the system from becoming too acidic, which is beneficial for the stability of the AD process and for increasing the rate of biogas production.

### 2.3 Laboratory setup and operational protocol

**FIGURE 1:**
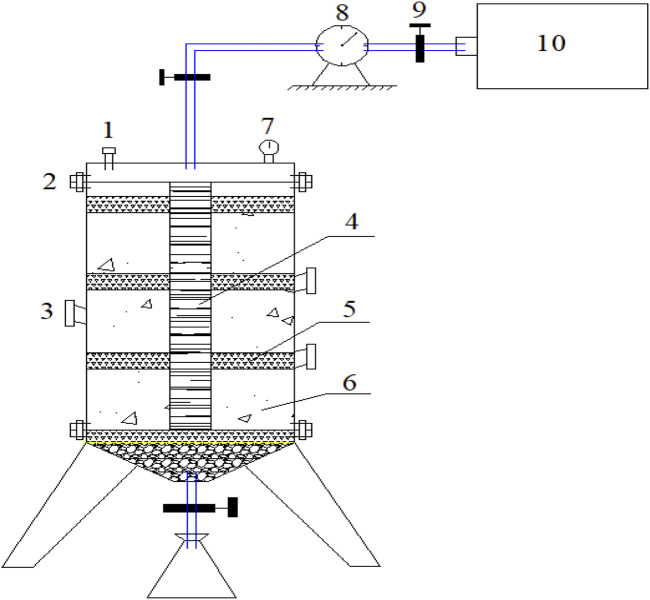
Schematic diagram of the experimental device. (1. safety valve; 2. flange plate; 3. sampling port; 4. PVC flower tube; 5. soil; 6. MSW; 7. pressure gauge; 8. flowmeter; 9. valve; 10. gas collecting bag).

The experimental reactor was a stainless steel cylindrical tank with a diameter of 30 cm and a central height of 60 cm, as shown schematically in Figure 1. A gas collection well, constructed from a 6-cm perforated plastic flower tube, was installed inside the reactor chamber. A leachate collection pipe with valves was located at the conical base of the column. The exhaust valve outlet was connected via a rubber tube to a gas collection bag and a wet gas meter. To regulate the temperature, a thermal regulation unit was installed along the exterior of the column to both heat the tank surface and control the waste temperature. The waste samples that were collected were first crushed into 2 cm–5 cm pieces and then thoroughly mixed with inoculated sludge. The mixed waste was then added to the reactor layer by layer. To create free drainage conditions, a 12-cm-thick layer of coarse gravel was compacted and placed on top of the gravel. Then, to avoid any blockage of the leachate collection pipe, a perforated metal plate was placed on top of the gravel. The solid waste was pressed and stratified every 19 cm, with each layer of waste being 17 cm thick, an MSW filling density of 800 kg/m^3^, and a 2-cm-thick layer of cover soil. In total, there were three layers of MSW and cover soil. After loading, the waste was sealed. The temperature of the reactor was maintained at 35 °C throughout the experiment.

### 2.4 Analytical methods

Carbon, hydrogen, nitrogen, and sulfur contents of the solid waste were measured using an elemental analyzer called Vario EL (Elementar, Germany). The volatile solids (VSs) were calculated based on ignition loss procedures. A wet gas flow meter was used in this study. Gas composition was analyzed using a “Fuli FL9510 gas” chromatograph with a thermal conductivity detector and a Porapak Q column (2 m length and 3 mm inner diameter) to measure the proportions of carbon dioxide (CO_2_) and CH_4_. Helium was used as the carrier gas. The percentages of CO_2_ and CH_4_ were determined relative to the reference gases.

By utilizing helium as the carrier gas and a Porapak Q packed column (2 m long and 3 mm internal diameter), the gas chromatograph equipped with a thermal conductivity detector was used to examine the gas composition, which included CH_4_ and CO_2_, after the gas volume had been computed using a wet gas flow meter. Then, the proportions of CH_4_ and CO_2_ were determined in relation to the reference gases.

A Mettler Toledo pH meter was used to assess the leachate’s pH, and a Shimadzu TOC-LCPH analyzer was used to quantify the TOC. Furthermore, a Shimadzu TNM-L TN analyzer was used to determine the leachate’s TN level. In addition, the Agilent GC-2010PLUSAF gas chromatograph was used to quantify VFAs, which include acetic, propionic, butyric, and valeric (HAc, HPr, HBu, and HVa) acids. The apparatus was equipped with a Chrompack DB-FFAP column (30 m × 0.25 mm, 0.25 µm), and nitrogen was used as the carrier gas. It was equipped with a flame ionization detector (FID).

### 2.5 DNA extraction and 16S rRNA gene sequencing

The leachate sample was filtered and fixed on a 0.2-μm pore-size filter membrane. For solid samples, 0.2 g of MSW samples and cover samples were directly weighed. DNA was extracted from all samples using the soil genomic DNA extraction kit (GeneMark) according to the manufacturer’s protocol. The concentration and purity of the extracted DNA were measured with an ultramicro-UV spectrophotometer (Implen Nanophotometer N60, Germany), and DNA integrity was assessed by 1% agarose gel electrophoresis containing 0.5 μg/mL ethidium bromide, which was run at 130 V for 20 min. DNA samples were stored at −20 °C in 1.5 mL centrifuge tubes.

The V3–V4 hypervariable region of the bacterial 16S rRNA gene was amplified using the following primers: ArBa515F (5′-GTGCCAGCMGCCGCGGTAA-3′) and Arch806R (5′-GGACTACVSGGGTATCTAAT-3′) on a GeneAmp 9700 thermal cycler (ABI, USA). The PCR mixture (20 μL) contained 4 μL of 5× FastPfu buffer, 2 μL of 2.5 mM dNTPs, 0.8 μL of each primer (5 μM), 0.4 μL of FastPfu polymerase, and 10 ng of template DNA. Thermal cycling conditions were as follows: 95 °C for 3 min; 30 cycles of 95 °C for 30 s, 55 °C for 30 s, and 72 °C for 45 s; followed by a final extension at 72 °C for 10 min. PCR products were separated on a 2% agarose gel, purified with the AxyPrep DNA Gel Extraction Kit (Axygen Biosciences, United States), and quantified using the QuantiFluor™-ST System (Promega, United States).

Paired-end sequencing (2 × 250 bp) was performed on the Illumina NovaSeq platform by Shanghai Meiji Biomedical Technology Co., Ltd. Raw sequencing data were processed and analyzed on the I-Sanger bioinformatics cloud platform. Sequences were clustered into operational taxonomic units (OTUs) at 97% similarity using USEARCH, and taxonomic assignment was conducted based on the SILVA database (release 138).

## 3 Finding and analysis

### 3.1 MSW gas generation and predictive modeling

Solid garbage filling was conducted on 17 October 2016. The gas production rate peaked at 3,022 mL/(d·kg)^−1^ after 3 days. Subsidence and extrusion released air from the solid waste’s pores, and the aerobic process decomposed any components that could decompose. Although the concentrations of O_2_ and CO_2_ in the gas mixture decreased and increased, respectively, nitrogen remained the principal component. No CH_4_ was generated during this initial period. A cumulative gas yield of 1,431 L, which was 57.3% of the total, was produced during the first 80 days of biogas production, making it the most productive part of the process. The breakdown of organic materials such as carbohydrates was the main cause of this, followed by facultative anaerobic fermentation and, eventually, production under the oxygen-limited condition. Production of CH_4_ occurred between days 60 and 200, and gas production was steady but sluggish. Perishable and biodegradable components of MSW, such as food scraps, had decomposed after 200 days. The system’s gas production is modest under these conditions. The total gas production of the reaction reached 2,498.61 L.

A measurable mathematical relationship between gas generation and time can be established by fitting the biogas production rate using a first-order kinetic approach based on the Scholl Canyon framework.

$$Q=kL_{0}e^{-kt},$$ (1)

where Q stands for the gas production rate in milliliters per kilogram per day (mL/(d.kg)^−1^), k represents the gas generation rate constant in decimal degrees, L_0_ represents the biogas production potential in milliliters per kilogram (mL/kg), and t represents the filling period in days.

**FIGURE 2:**
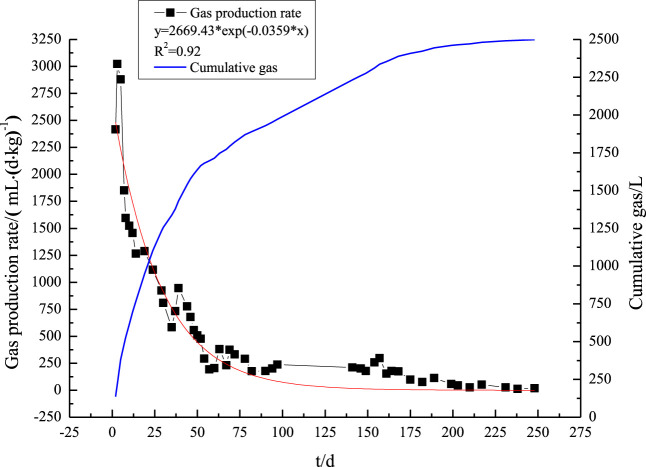
Trend chart of biogas production rates.

Figure 2 shows the results of applying exponential regression using Equation 1 to the observed gas production rate over a 250-day period. With a strong correlation coefficient of 0.92, the research findings demonstrated that the gas production rate from MSW AD decreased exponentially. The outcomes reveal that the Scholl Canyon method correctly depicts the trend of gas generation over time. Both a predicted biogas yield of 74.36 L kg^-1^ of waste and a rate constant of 0.0359 days^-1^ were derived from the model fitting. These findings are in agreement with those of Kjeldsen et al. (2002), who found that 96.31 L kg^-1^ of gas was produced annually by landfills from food and paper waste.

### 3.2 Impact of leachate properties on methane production

**FIGURE 3:**
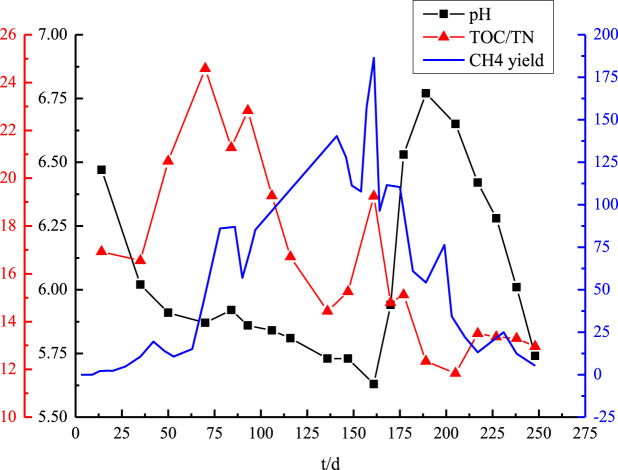
Trend chart of pH value and TOC/TN.

Figure 3 shows the changes in the pH, TOC, TN, and CH_4_ generation rate over time. Similar to what has been observed in AD bioreactors using leachate recirculation (He et al., 2016), the leachate pH fluctuated throughout the experiment, increasing and decreasing repeatedly. In particular, Figure 3 shows that the pH decreased from 6.47 to 5.91 in the initial 50 days. This decrease was primarily caused by the hydrolysis and acidification of easily degradable waste components during the initial stage of digestion (He et al., 2011), which in turn led to the buildup of organic acids (Kayhanian, 1999). From the 50th to the 160th day, the pH remained relatively constant, falling somewhere between 5.91 and 5.63. Microbes used the by-products of acidification and hydrolysis to fuel cell development and CH_4_ synthesis, which led to steadily increasing CH_4_ yields throughout the production phase. Therefore, the pH remained relatively stable, and no sustained acidification occurred.

During the 160th day, the pH dropped to its lowest point at 5.63 and the CH_4_ outputs reached a peak of 186.45 mL/(d·kg)^−1^. After that, there was a marked increase in the pH, which peaked at 6.77, at roughly 30 days after the CH_4_ peak. The accumulation of metabolic by-products during methanogenesis inhibited the growth of the CH_4_-producing bacteria. The continued fermentation of cellulose and proteins also caused the pH to fall again as organic acid buildup resumed. The pH of leachate typically peaks at the same time as CH_4_ production, which can help indicate the timing of methanogenesis.

The TOC/TN concentration showed two peaks throughout the experiment. At the start of the reaction, the TOC/TN increased from 16.55 to 24.58. The first peak occurred on the 70th day, which was caused by carbohydrate hydrolysis, and then the ratio decreased. The sub-peak occurred on the 160th day, when protein and hemicellulose degraded, corresponding to the peak of CH_4_ production. At the methanogenic stage, TOC/TN values fluctuated in the range of 12.33 ∼ 24.58.

The progressive reduction in the TOC/TN ratio took place in the final stage of CH_4_ production, mostly due to nitrogen accumulation (Sakita et al., 2016). Ammonia is the most abundant type of nitrogen in the leachate, which is primarily generated from the breakdown and modification of proteins (Kjeldsen et al., 2002). Kjeldsen et al. (2002) pointed out that even after long periods of CH_4_ generation, ammonia concentrations remain high, which could lead to environmental problems in the future. Due to the sensitivity of AD systems to ammonia–nitrogen, methanogens are inhibited by high concentrations of ammonia (Angelidaki and Ahring, 1986; Desloover et al., 2012). As a result, the observed decrease in gas production during the AD of MSW can be largely attributed to the inhibition caused by elevated levels of ammonia. One measure of the methanogenic system’s stability during MSW degradation is the TOC/TN ratio in the leachate. The AD process stability is maintained, allowing for normal gas generation, when the TOC/TN ratio exceeds 13. On the other hand, if the TOC/TN ratio falls below 13, it indicates that ammonia may slow down the process and render the fermentation system unstable (Wang et al., 2009).

The amount of VFAs, an intermediate metabolite in AD that indicates the extent of substrate breakdown and the efficiency of CH_4_ generation (Patil and Deshmukh, 1996), is an important indicator of these processes. In an AD system, methanogens can directly use intermediates gases, such as hydrogen, carbon monoxide, HAc, methanol, and formic acid, to produce CH_4_. Nevertheless, acidification, caused by the accumulation of long-chain fatty acids, negatively affects CH_4_ generation. The growth of methanogens is strongly reduced by high quantities of propionic acid (De Bok et al., 2001).

**FIGURE 4:**
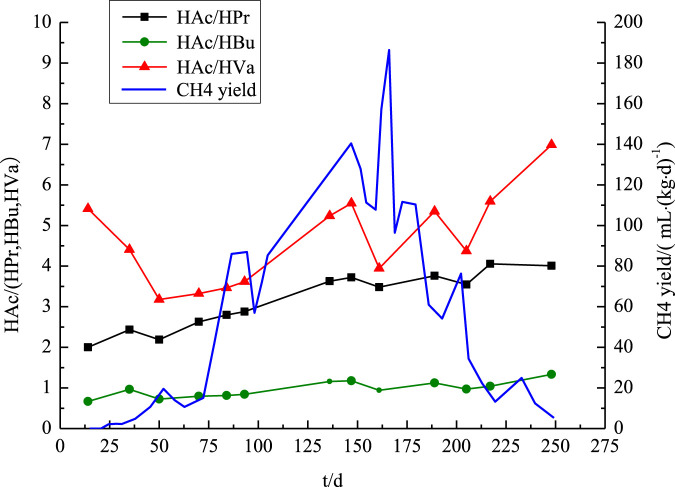
Trend chart of VFAs.

HAc, HPr, HBu, and HVa concentrations were determined in this study by analyzing the leachate samples (Supplementary Table S1). In order to reduce the possibility of sampling mistakes, the CH_4_ yield rates were examined in conjunction with the patterns in fluctuation of the ratios (HAc/HPr, HAc/HBu, and HAc/HVa), as shown in Figure 4. According to the figure, at the beginning, HAc/HBu was less than 1, HAc/HPr was greater than 1, and HAc/HVa was higher than HAc/HPr. Hence, HBu > HAc > HPr > HVa was the order of the first VFAs concentrations in the leachate.

There was an increase in the HAc/HBu and HAc/HPr ratios during the CH_4_ production stage, which may indicate that the bacteria consumed a wide variety of acids at once while converting HAc into CH_4_. Evidence suggests that propionate can be converted into HAc and HBu through six-carbon intermediates (Zhang B. et al., 2010). During the peak of CH_4_ production, the HAc/HVa ratio showed an upward fluctuation with a noticeable trough, indicating unique inflection points that can be utilized to predict the time of CH_4_ synthesis driven by HAc.

Apart from HAc/HVa, which sharply decreased from 5.41 to 3.17 after 50 days, the two other ratios remained relatively constant. This indicates that the hydrolysis–acidification step was the peak period for HVa synthesis, likely aided by easily biodegradable components in MSW. All three ratios (HAc/HBu, HAc/HPr, and HAc/HVa) increased from their starting points. These results showed that the accumulation of inhibitory metabolites effectively suppressed CH_4_ production by suppressing the activity of methanogenic acetogens, as evidenced by the elevated HAc concentrations.

### 3.3 Microbial composition and population

#### 3.3.1 Microbial composition diversity

Community diversity can reflect the structural stability of microbial communities (Spang et al., 2015). Three stages of landfill leachate samples (at the hydrolytic acidification period A, at the CH_4_ production peak period B, and at the end of CH_4_ production C), MSW samples, and cover soil samples at the end of the experiment were analyzed by 16S rRNA gene tag pyrosequencing for community diversity assessment. Table 3 shows the total number of sequences and the diversity index of the community. The coverage of the five samples was more than 99%, indicating that the sample sequencing results represented the real conditions of the sample. Comparing the a-diversity index (Chao index) of the samples, it was found that the microbial diversity of the MSW samples at the end of the reaction was the most abundant, followed by the cover sample. Leachate samples at the initial stage of CH_4_ production had the highest microbial diversity.

**TABLE 3:** Scheduling consequences and alpha diversity assessments.

Samples	Number of sequences	OTUs	Alpha diversity^a^	Coverage (%)
A	31,629	30,864	32.00	99.99
B	40,401	39,105	30.00	99.99
C	43,784	42,424	29.25	99.99
MSW	41,206	39,438	46.50	99.99
Cover	42,796	40,482	36.78	99.99

^a^ Alpha diversity as measured by the inverse Chao’s index. Higher values indicate higher diversity.

#### 3.3.2 Microbial profile characteristics

**FIGURE 5:**
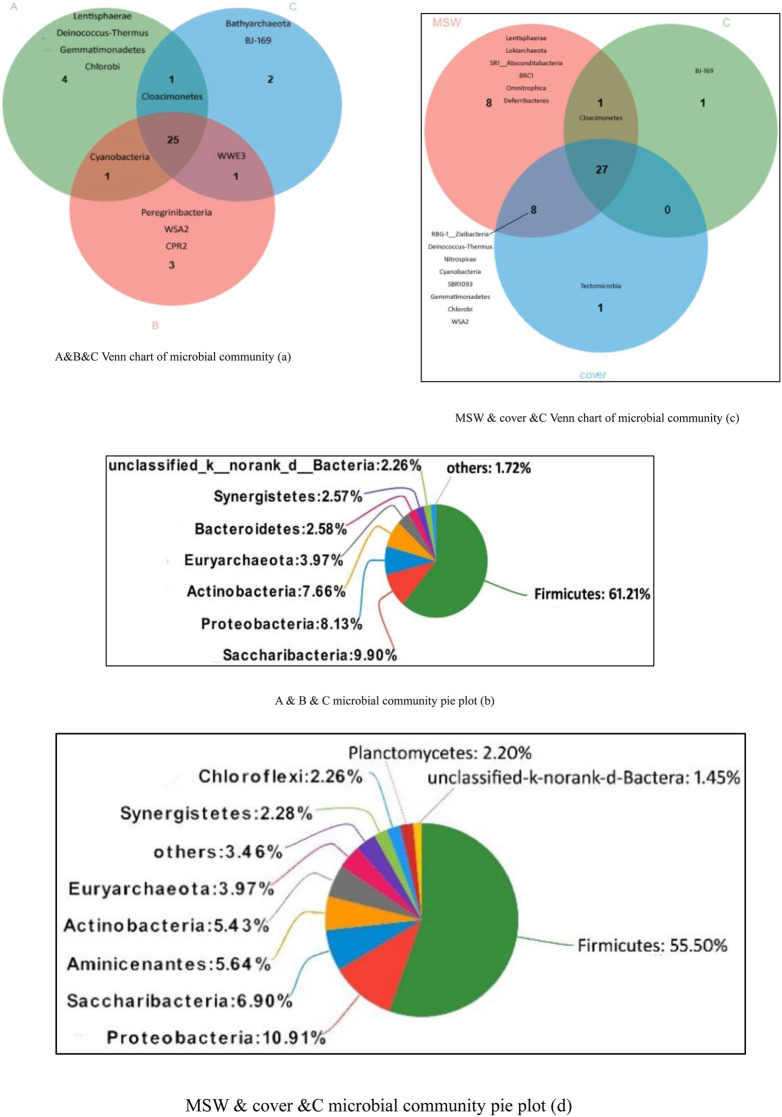
Venn chart and pie plot of the microbial community based on the phylum level. A, B, C: Leachate samples from the landfill at the hydrolytic acidification stage **(a)**, methane production peak **(b)**, and end of methane production **(c)**, respectively. MSW: municipal solid waste sample collected at the end of the experiment. Cover: soil cover sample collected at the end of the experiment. **(d)** MSW & cover & C microbial community pie plot.

The microbial community structure changed during different stages of MSW digestions. The Venn chart and pie plot of microbial community based on the phylum level are shown in Figure 5. The Venn chart shows the number of common and unique species in each sample. Figure 5 shows that the number of species in the leachate during the three periods was similar, with 31, 30, and 29 species at different fermentation times, respectively. The number of microbial populations in the MSW and cover samples was higher than that in leachate samples, which were 44 and 36, respectively. The results were consistent with the diversity index of the community in Table 3. The number of microbial populations was the highest at the initial fermentation stage in which the biomass communal synergism and the biochemical reaction were more active. Among them, *Lentisphaerae*, *Deinococcus–Thermus*, *Gemmatimonadetes*, and *Chlorobi* are special to this stage. The 25 common microbial species in the leachate samples (relative abundance >1%) and their relative abundance are shown in Figure 5b. Notably, *Nitrospira* was not detected in all three leachate samples. *Nitrospira* was used as a nitrifier to oxidize nitrite to nitrate. In the absence of *Nitrospira*, the ammonia–nitrogen/nitrate/nitrite cycle was interrupted. The experimental results showed that targeted microbial inoculation was a good way to improve AD performance; in particular, inoculation using bio-based treatments including *Nitrospira* markedly improved the stability of AD and increased the efficiency of landfill leachate treatment. Figure 5c shows the Venn chart at the phylum level of leachate samples, MSW sample, and cover sample at the end of the experiment. The *Lokiarchaea* archaea were detected in the MSW sample. Members of this group contain some of the genes encoding the proteins and are considered among the closest prokaryotic species of eukaryotes (Wilson et al., 2014). Therefore, *Lokiarchaea* are regarded as a bridge between prokaryotes and eukaryotes (Kim et al., 2015). *Lokiarchaea* are usually found in nutrient-deprived deep-sea sediments and have low cell counts, which makes it difficult to obtain the desired amount of cells through culture in the laboratory. A unique microorganism in the cover sample was *Tectomicrobia*, which is identified as a biochemically talented phylum equivalent to actinomycetes (Li et al., 2017). Adding soil for intermediate coverage during landfill disposal can introduce dominant species, which markedly enhances the rate of waste degradation. In addition, it can be seen from the OTUs table that *Nitrospira*, which includes five species, mainly existed in the cover sample, and the abundance of *bacterium_WX65* was the largest, whereas there was only one species in the MSW sample, and its abundance was smaller. This indicates that the cover layer is the main location for the nitrification system. The absence of *nitrosobacteriosis* leads to ammonia accumulation in the system, which inhibits the microbial activity and is not conducive to digestion and gas production of the system.

**FIGURE 6:**
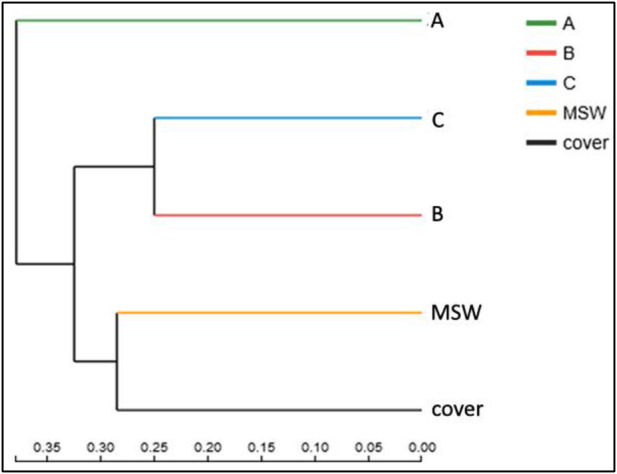
Hierarchical clustering tree of the microbial community on the OTU level. A, B, C: Leachate samples from the landfill at the hydrolytic acidification stage (A), methane production peak (B), and end of methane production (C), respectively. MSW: municipal solid waste sample collected at the end of the experiment. Cover: soil cover sample collected at the end of the experiment.

Figure 6 shows a hierarchical clustering tree of microbial community at the OTU level. Cluster analysis showed that the MSW sample and leachate samples had a close genetic relationship, and the longer the reaction period was, the higher the similarity became. The leachate sample was a product of MSW AD, and its microbial species and abundance were relatively close. The community composition of the leachate samples can reflect the community structure of the MSW in the system to some extent, which could be used for the prediction of the MSW digestion process.

**FIGURE 7:**
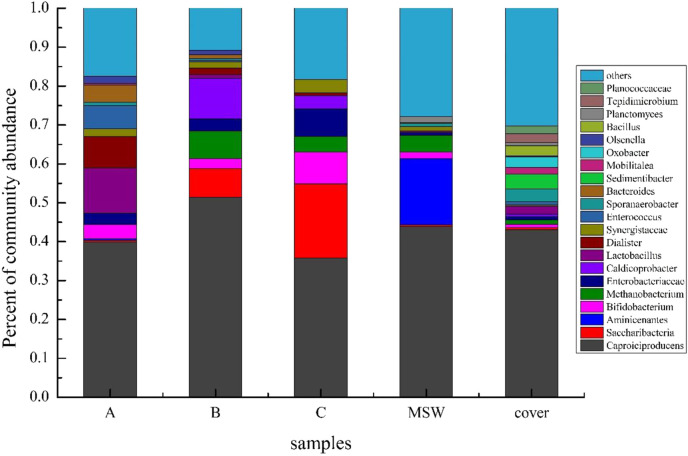
Microbial community bar-plot on the genus level. A, B, C: Leachate samples from the landfill at the hydrolytic acidification stage (A), methane production peak (B), and end of methane production (C), respectively. MSW: municipal solid waste sample collected at the end of the experiment. Cover: soil cover sample collected at the end of the experiment.

The microbial community (genus level, relative abundance greater than 1%) is illustrated in Figure 7, which shows that Caproiciproducens constitutes 38.5%–51.4% of the total OTUs across all five samples. We differentiated the seeding sludge and MSW-derived communities based on OTU composition, which allowed us to separate the microbial populations. Although Caproiciproducens, a member of the genus *Clostridium* within the phylum Firmicutes, was present in both the inoculum and MSW, its relative abundance was much higher in the inoculated sludge, indicating that the inoculum strongly contributed to the enrichment of this bacterial species. Mali et al. (2012) reported that Caproiciproducens is a Gram-positive, strictly anaerobic bacterium that thrives at 35 °C–45 °C and pH 6.0–8.0 based on its isolation from wastewater treatment plants. In addition, the bacteria can use galactose as the carbon source, whose anaerobic fermentation metabolites include hydrogen, CO_2_, ethanol, HAc, HBu, and caproic acid (Boone and Whitman, 2015). These results indicated that the fermentation system produced hydrogen and that the acid production process was stable. This stage was not the limiting step of the whole digestion process. *Methanobacterium* belongs to the *Euryarchaeota*. One way to quantify the activity of CH_4_ generation in AD systems is by looking at the relative abundance of methanogens. In the leachate sample (B), the relative abundance of methanogens reached 7.02% and that of methanogens from the leachate sample (C) decreased to 3.94%. However, in the leachate sample (A), the relative abundance of methanogens was low, less than 1%, indicating that methanogenic archaea were gradually domesticated and enriched with the process of waste fermentation. The relative abundance of methanogens in the MSW sample was higher than that in the cover sample, indicating that methanogens were more likely to attach to nutrient-rich litter particles. In addition, by comparing the dominant species (relative abundance greater than 1%), more microbial species were detected in the cover sample than in the MSW sample, indicating that the intermediate cover effectively increased the microbial biomass (Smith and Ingram-Smith, 2007) in the process of landfill disposal. The intermediate cover layer acted as a biochemical reaction layer, which promoted AD and enhanced the stability of the system.

**FIGURE 8:**
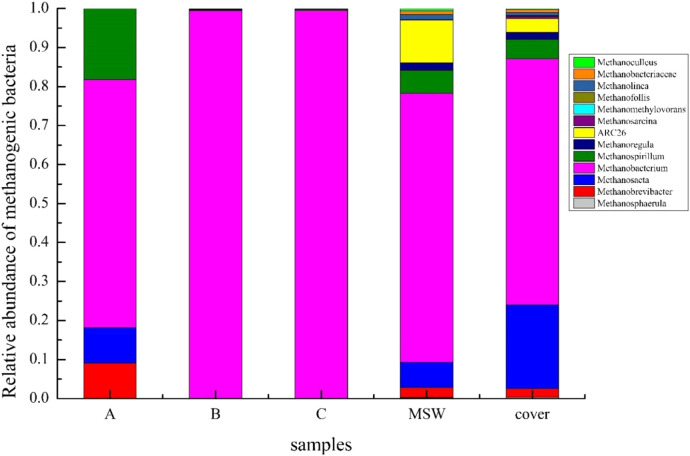
Methanogen community structure bar-plot on the genus level.

Figure 8 shows the methanogen community structure bar-plot at the genus level. As shown in Figure 8, 13 kinds of methanogenic archaea were detected, among which *Methanobacterium*, *Methanosaeta*, and *Methanospirillum* occupied a relatively large proportion. *Methanobacteria* in the MSW and cover samples was more abundant than that in leachate samples. More toxic substances accumulated in the leachate, which inhibited the growth of methanogens that were less tolerant to toxicity. *Methanobacterium* accounted for more than 99% of the leachate samples (B and C), and it remained dominant in other samples. It is a strictly anaerobic bacterium; some members of this genus can utilize formate, whereas others live exclusively through the reduction of CO_2_ with hydrogen. The reason why *Methanobacterium* is widespread in the system may be attributed to its ability to metabolize ammonia and N_2_O_4_ as nitrogen sources (Ferry et al., 2015) and its strong resistance to ammonia toxicity. At the same time, this demonstrated that from another perspective, that at the end of the fermentation system, the system exhibited an accumulation of ammonia due to the absence of nitrifying bacteria, which resulted in the inactivation of other methanogenic archaea. *Methanosaeta* was previously considered an acetate methanogen, and recent studies have identified a thermophilic gene and a gene encoding CO_2_ reductase in *Methanosaeta* (Ferry et al., 1974). The competitive advantage of *Methanosaeta* was enhanced by its strong tolerance to temperature and a more diverse metabolic pathway. The metabolic pathway of *Methanospirillum* involves the use of formate or hydrogen and CO_2_ as substrates for cell proliferation and CH_4_ production (Ferry et al., 1974) rather than acetate, pyruvate, methanol, ethanol, and benzoate. In conclusion, the main methanogenic archaea in the reactor were hydrogen-utilizing methanogenic archaea that were more environmentally adaptable than acetate-utilizing methanogenic archaea. The lack of acetate-utilizing methanogens led to the end of gas production.

## 4 Conclusion

Consequently, the gas production pattern in the AD system of MSW exhibited an exponential fall after an initial buildup, as per the Scholl Canyon model. The model fitting indicated that MSW digestion could produce biogas at a rate of 0.0359 m^3^/d, with a yield of 74.36 L/kg. This number did not take the seed sludge’s contribution into consideration; it represented the overall biogas output. Although some information was provided by the leachate pH, the AD system’s noticeable lag made pH readings a poor indicator of CH_4_ output. With time, the concentration of TN in the leachate increased, indicating nitrogen accumulation. This, in turn, inhibited gas formation because of the high quantities of ammonia.

The leachate TOC to TN ratio also served as a good measure of system stability. In particular, a TOC/TN ratio greater than 13 indicated system stability and continuous gas production; a ratio lower than 13 stated instability and decreased gas output. It was possible to predict when CH_4_ will be produced as the HAc/HVa ratio in the leachate showed large oscillations with obvious inflection points.

A comparative analysis of microbial community distribution between the MSW and cover samples demonstrated that the intermediate cover layer acted as a key biochemical reaction zone, where its greater microbial diversity promoted AD and thereby enhanced the system stability.

Likewise, with longer reaction times, genetic tests showed a higher link between MSW and leachate samples. Nevertheless, *Nitrospira*, which is commonly detected in the cover layer where nitrification occurs, was absent from both the leachate and MSW samples. The cover layer harbored five *Nitrospira* species, indicating that it serves as the primary site for nitrification and plays a critical functional role in the system’s nitrogen cycle.

## Data Availability

The data presented in the study are deposited in the National Tibetan Plateau Data Center repository, accession number https://data.tpdc.ac.cn/en/disallow/9955716e-2a4a-403f-8182-b56f33f1d0f1
