# Supplementary material for: Biogas production and microbial profile estimation in bioreactor landfills
Source: Front Chem. 2026 Apr 7;14:1742729. doi: 10.3389/fchem.2026.1742729 (PMC13095782; doi:10.3389/fchem.2026.1742729)
Supplement: Supplementary file 1 [file Table1.docx]

Supported Table S1

Concentrations of valeric acids (HAc, HPr, HBu, and HVa)

| Days | HAc | HPr | HBu | HVa |
| --- | --- | --- | --- | --- |
| 14 | 596.274 | 297.5449 | 890.5593 | 110.1567 |
| 35 | 585.942 | 240.5384 | 605.8753 | 132.9438 |
| 50 | 511.371 | 233.4452 | 704.1059 | 160.8125 |
| 70 | 537.424 | 204.7376 | 675.818 | 161.5934 |
| 84 | 565.601 | 202.2221 | 697.6699 | 163.3226 |
| 93 | 564.034 | 195.7629 | 667.1327 | 155.954 |
| 136 | 564.034 | 155.4421 | 487.7626 | 107.6752 |
| 147 | 514.592 | 138.3275 | 436.7797 | 92.81628 |
| 161 | 29005.5 | 8327.209 | 30778.33 | 7349.658 |
| 189 | 29297.1 | 7781 | 26019.89 | 5479.443 |
| 205 | 25568.6 | 7207.234 | 26304.06 | 5850.322 |
| 217 | 26871.2 | 6629.413 | 25824.53 | 4803.515 |
| 248 | 29820.8 | 7440.876 | 22359.45 | 4268.694 |
